# Detecting Vehicle Loading Events in Bridge Rotation Data Measured with Multi-Axial Accelerometers

**DOI:** 10.3390/s22134994

**Published:** 2022-07-02

**Authors:** Alan J. Ferguson, Roger Woods, David Hester

**Affiliations:** 1 School of Electronics, Electrical Engineering and Computer Science, Queen’s University Belfast, Ashby Building, Stranmillis Road, Belfast BT9 5AG, UK; aferguson29@qub.ac.uk; 2 School of Natural and Built Environment, Queen’s University Belfast, David Keir Building, Stranmillis Road, Belfast BT9 5AG, UK; d.hester@qub.ac.uk

**Keywords:** event detection, bridge structural health monitoring, rotation measurement, generalised variance

## Abstract

Structural Health Monitoring (SHM) is critical in the observation and analysis of our national infrastructure of bridges. Due to the ease of measuring bridge rotation, bridge SHM using rotation measurements is becoming more popular, as even a single DC accelerometer placed at each end of span can accurately capture bridge deformations. Event detection methods for SHM typically entail additional instrumentation, such as strain gauges or continuously recording video cameras, and thus the additional cost limits their utility in resource-constrained environments and for wider deployment. Herein, we present a more cost-effective event detection method which exploits the existing bridge rotation instrumentation (tri-axial MEMS accelerometers) to also act as a trigger for subsequent stages of the SHM system and thus obviates the need for additional vehicle detection equipment. We show how the generalised variance over a short sliding window can be used to robustly discriminate individual vehicle loading events, both in time and magnitude, from raw acceleration data. Numerical simulation results examine the operation of the event detector under varying operating conditions, including vehicle types and sensor locations. The method’s application is demonstrated for two case studies involving in-service bridges experiencing live free-flow traffic. An initial implementation on a Raspberry Pi Zero 2 shows that the proposed functionality can be realised in less than 400 ARM A32 instructions with a latency of 47 microseconds.

## 1. Introduction

Bridges form a key part of every nation’s critical national infrastructure, carrying thousands of vehicles and providing essential links for citizens and businesses. However, ageing, deterioration and extreme events threaten these key assets, resulting in economically damaging bridge closures or even collapses [1]. Consequently, many different bridge structural health monitoring (BSHM) approaches have been proposed, some using strain [2], displacement [3], vibration [4] or acceleration [5] in order to detect damage or deterioration in the bridge response. Recently, rotation-based BSHM has been shown to offer a more practical approach for sensing short to medium bridge deflections under loading [6].

Complex state-of-the-art BSHM solutions have been developed for long-span bridges, such as the Queensferry Crossing [7] and Hong Kong–Zhuhai–Macau [8] bridges, thus allowing continuous monitoring. However, for the vast majority of the bridge stock, their structural condition is only assessed by infrequent, human-visual inspection, and continual, sensor-based monitoring is limited by practical issues such as limited power budget and communications capacity. The ideal, therefore, is to provide autonomous BSHM systems that can enable reliable, long-term rotation monitoring [9] in these resource-constrained environments. For example, BSHM needs only to record and analyse the rotation response when the bridge undergoes external loading as a result of vehicles crossing. For the low traffic volumes experienced at night or in rural settings, this results in a rotation response characterised by short windows of signal, corresponding to vehicle crossings, dispersed in often long periods of noise.

Furthermore, once these individual events are identified, the ability to distinguish between truck loading events and car loading events is important. This is because bridges are designed to support heavy truck loading; therefore, cars are typically not heavy enough to generate significant deformations in the bridge. Hence, the deformation response signals due to cars often contain little information useful for bridge SHM. This is particularly true of approaches that are interested in the static component of the bridge response, e.g., maximum displacement or maximum rotation.

Consequently, a method that can quickly and easily distinguish truck loading events from car loading events or noise would be very useful as this can avoid using scarce power and communications resources to record and transmit sensor signals data containing little or no useful information. Thus, BSHM solutions would ideally employ vehicle detection systems, preferably including vehicle classification, therefore allowing the system to obtain just these regions of interest when there was load on the bridge and thereby concentrating data capture efforts.

Inductive-loop-based vehicle detectors [10] and magnetometers [11] can potentially provide some details of vehicle classification, but it can be costly to retrofit them to existing bridges. On the other hand, computer-vision-based techniques [3] do not need to contact the structure and can be deployed relatively quickly. Non-contact optical techniques can be used to measure the 3D profile of a vehicle [12] or to detect axles [13]. Wi-Fi channel state information [14] has also been used to provide similar non-contact traffic monitoring. Strain measurements [15] and vibration sensing [16] have been shown to provide vehicle detection and classification information.

A major shortcoming of these approaches is that their practical deployment requires that additional instrumentation, e.g., induction loops, be installed on the structure under test; with 118,000 road and rail bridges in Great Britain alone, this would be highly costly to implement as a widely available solution. Moreover, with variance in communications bandwidth and reliable energy supply in these locations, the design needs to effectively use available resources and capture relevant data in a more efficient manner. Here, we employ a ‘more for less’ approach and obtain the necessary vehicle detection and classification information from the data provided by the existing rotation monitoring systems. This work forms part of a larger collaboration effort to provide enhanced BSHM systems involving both the electronics and civil engineering research groups at Queen’s University Belfast.

**Figure 1:**
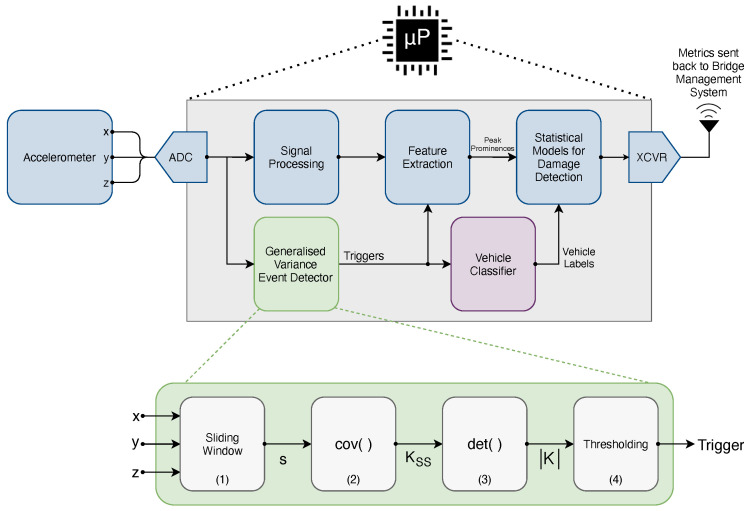
Block diagrams showing the overview of a typical rotation monitoring system (blue) with the added event detector functionality (green) and vehicle classifier (purple).

In this work, we examine an extension to a typical, current BSHM sensing node design (Figure 1) to investigate if vehicle detection can be performed without the need for additional instrumentation. To achieve this, we exploit the existing instrumentation, in this case, tri-axial MEMS accelerometers, which are widely employed within BSHM. Using the raw data from these accelerometers, we show that it is possible to detect regions of interest corresponding to vehicle crossings by tracking the increases in the generalised variance of the sample covariance matrix (computed between the channels of the muli-axial accelerometer) over a sliding window. Covariance-based event detection methods have been widely employed in other areas, including power systems measurement [17], video surveillance [18] and geophysics [19]. The approach is based on the observations that (a) during vehicle crossings, the measured multi-axial acceleration signals will exhibit covariance due to the systemic effect of the vehicle loading, and (b) in the periods between crossings, a rotation monitoring system only measures noise. Providing reliable tracking capability to an existing monitoring resource thus provides a high potential for a highly cost-effective approach. The validity and application is demonstrated here.

The paper describes the creation of an event detection method that builds event detection capability into existing bridge rotation monitoring instrumentation and applies it to full-scale bridge data. Our main contributions are as follows:

- The creation of a low-cost detection method based on the generalised variance between tri-axial accelerometer signals, which can be implemented cost-effectively by using available resources and thereby avoiding the additional cost and energy usage of dedicated traffic-detection hardware.
- Detailed numerical simulations have been undertaken to show that the method is robust to the effects of sensor noise and rotation signal variability due to varying sensor location, vehicle speeds and vehicle type, as well as the effect of sliding window lengths.
- Applications of the approach to two case studies demonstrating successful detection of vehicle events using end of span accelerometer signals collected on in-service highway bridges.

The rest of this paper is structured as follows: Our rotation event detector method is set out in Section 2; Section 3 describes the numerical models used in this work; Section 4 presents our numerical simulations and results for the proposed event detector; Section 5 outlines two case studies using the event detector method on in-service bridges with live traffic; and finally, Section 7 concludes this paper.

## 2. Event Detection Method

The purpose of this work is to develop an event detection method which leverages the existing bridge rotation instrumentation (tri-axial MEMS accelerometers) and that can run on low-resource sensor nodes. In Figure 1, the blue blocks represent our existing rotation monitoring approach, described in [9], with the resultant damage detection metrics sent back to the bridge management system to inform operational decision making.

The proposed event detector block (shown in green) takes the raw digitised acceleration samples and provides trigger signals to indicate regions of interest to the feature extraction stage and our external vehicle classifier system. Previously, this vehicle classifier operated continually on video data to perform real-time object detection. Instead, this can be implemented more efficiently by capturing short bursts of frames using the event detector trigger and batch-processing these. This reduces the system’s power consumption by possibly avoiding the need for video capture altogether and spending less time running the computationally intensive object detector code. Whilst the system has been incorporated into our detector system for demonstration, the technique has general applications to any system that uses accelerometers.

The lower part of Figure 1 shows the four stages of our event detector (annotated (1) to (4)). In the first block, a sliding window stores the most recent raw digital acceleration samples in a circular buffer. Block 2 computes the covariance matrix, $K_{\mathrm{SS}}$, over the sliding window. Block 3 calculates the generalised variance, $\det\left(K_{\mathrm{SS}}\right)$. Finally, the last block (4) implements a threshold function to generate the trigger signal output. The event detector block has been implemented using 380 ARM A32 instructions (Algorithm 1). Running on a Raspberry Pi Zero 2, this single-threaded implementation has a latency of 47 μs.
**Algorithm 1** Generalised Variance-based Event Detector**Intput**$N_{w}$ (sliding window length in samples),$\left\{a_{x},a_{y},a_{z}\right\}$ (new acc. samples),thres (detector threshold level)**Output**trig (region of interest trigger)**Initialisation**Set up circular buffers $B_{x},B_{y},B_{z}$ with lengths, $N_{w}$**Runtime**Push $a_{x},a_{y},a_{z}$ into buffers $B_{x},B_{y},B_{z}$.Recompute covariance matrix, $K_{\mathrm{ss}}$, over buffers.Calculate generalised variance, $\det(K_{\mathrm{ss}})$.Update trig based on $\det(K_{\mathrm{ss}})$ and thres.

The remainder of this section provides more details on the operating principles of our proposed event detector system (lower part of Figure 1). For the single tri-axial accelerometer that we use here, the noise-free acceleration, a, observed by the accelerometer consists of: (i) the rotated Earth’s gravity vector, g, in the reference frame of the accelerometer and (ii) the translational acceleration, $a_{b}$, associated with both the bridge’s deflection under load and modal vibrations.

We first define the global co-ordinate system, x,y,z, which corresponds to the longitudinal, transverse, and vertical axes, respectively, of the bridge deck at rest. The X,Y,Z axes of the tri-axial accelerometer’s reference frame are assumed to be co-linear with the corresponding global x,y,z axes. We then denote the improper Euler angles ψ,θ,φ about x,y,z, respectively, for the rotation of the accelerometer due to the bridge’s deflection under load. From these angles, the rotation matrices for the accelerometer’s reference frame, $R_{x}\left(\psi \right),R_{y}\left(\theta \right),R_{z}\left(\varphi \right)$, about x,y,z, respectively, are formed. Thus, the noise-free acceleration components, $a={\left[a_{X},a_{Y},a_{Z}\right]}^{⊤}$, corresponding to the rotation of the gravity vector, g, and translational acceleration, $a_{b}$, in the reference frame of the accelerometer are obtained by:

(1) $$\begin{array}{cc} a & =R_{z}\left(\varphi \right)\times R_{y}\left(\theta \right)\times R_{x}\left(\psi \right)\times \left(g+a_{b}\right) \end{array}$$

We consider an additive noise model, i.e., s=a+n, wherein the measured signal, s, is a linear combination of the true acceleration, a, and a random noise variable, $n={\left[n_{X},n_{Y},n_{Z}\right]}^{⊤}$, each with finite variances and means.

A rectangular sliding window (block 1 in Figure 1) is used to sample the *m* most recent measured signal data points, i.e., $S=\left[s_{1},\cdots ,s_{m}\right]$.

The sample covariance matrix (block 2 in Figure 1), $K_{\mathrm{SS}}$, over the sliding window, *S*, is formed by:

(2) $$K_{\mathrm{SS}}{}_{i,j}=\mathrm{cov}\left[s_{i},s_{j}\right]=\mathrm{cov}\left[a_{i}+n_{i},a_{j}+n_{j}\right].$$

Due to the bilinearity of covariance:

(3) $$K_{\mathrm{SS}}{}_{i,j}=\mathrm{cov}\left[a_{i},a_{j}\right]+\mathrm{cov}\left[a_{i},n_{j}\right]+\mathrm{cov}\left[n_{i},a_{j}\right]+\mathrm{cov}\left[n_{i},n_{j}\right].$$

Independence is assumed between a and n, i.e., $\mathrm{cov}\left[a_{i},n_{j}\right]=\mathrm{cov}\left[a_{j},n_{i}\right]=0$, but not the individual noise components $n_{X}$, $n_{Y}$ and $n_{Z}$ due to potential common mode noise. Thus:

(4) $$K_{\mathrm{SS}}{}_{i,j}=\mathrm{cov}\left[a_{i},a_{j}\right]+\mathrm{cov}\left[n_{i},n_{j}\right].$$

By comparing with Equation (2), we determine that $K_{\mathrm{SS}}$ is a linear combination of the covariance matrices, $K_{\mathrm{AA}}$ and $K_{\mathrm{NN}}$, for the acceleration of gravity and noise respectively, i.e.,

(5) $$K_{\mathrm{SS}}=K_{\mathrm{AA}}+K_{\mathrm{NN}}.$$

The generalised variance [20], $\det\left(K_{\mathrm{SS}}\right)$, is then taken (block 3 in Figure 1) to provide an one-dimensional measure of the sample covariance matrix, $K_{\mathrm{SS}}$. As the covariance matrix is positive semi-definite [21], the Minkowski determinant theorem [22] implies:

(6) $$\begin{array}{c} \det{\left(K_{\mathrm{AA}}+K_{\mathrm{NN}}\right)}^{\frac{1}{n}}\geq \det{\left(K_{\mathrm{AA}}\right)}^{\frac{1}{n}}+\det{\left(K_{\mathrm{NN}}\right)}^{\frac{1}{n}}. \end{array}$$

Ambient traffic loading comprises a sequence of discrete events caused by heavy moving loads (e.g., lorries and buses) crossing the bridge at random time intervals. As the bridge is assumed to remain at rest when not experiencing loading, i.e., a=0, when no loading events are within the sampled period:

(7) $$a=0\Rightarrow K_{\mathrm{AA}}=0_{}∴K_{\mathrm{SS}}=K_{\mathrm{NN}}.$$

Based on Equations (6) and (7) and assuming the noise process remains stationary over the period of interest, then $\det\left(K_{\mathrm{AA}}+K_{\mathrm{NN}}\right)>\det\left(K_{\mathrm{NN}}\right)$, i.e., an increase in $\det\left(K_{\mathrm{SS}}\right)$ occurs during a vehicle crossing event. This increase in generalised variance is turned in a trigger signal by the thresholding block in Figure 1.

## 3. Numerical Models

To demonstrate the operation of the proposed event detector, a finite-element (FE) model is developed to simulate a bridge undergoing traffic loading, with particular attention given to the response near the end of span supports, and this is described in Section 3.1. An accelerometer signals model, presented in Section 3.2, is then used to make the structural dynamics data obtained from the FE model more representative of measured bridge rotation data.

### 3.1. Bridge Finite Element Model

**Figure 2:**
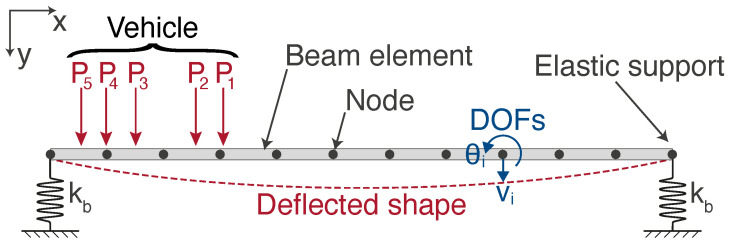
Sketch of 2D discretised bridge model used.

Typically, significant levels of rotation are only observed about the transverse axis, i.e., $\left|\theta \right|>0$ and $\left|\psi \right|\approx \left|\varphi \right|\approx 0$, and similarly, displacements are only significant in the vertical axis. Figure 2 shows the two dimensional bridge FE model used to represent a 25 m long by 14.6 m wide bridge. It is based on previous work described in [5], which gives a fuller description of the model. The one modification from the previous FE model is in the boundary conditions, where the supports are no longer idealised as being infinitely stiff in the vertical degree-of-freedom. It is widely understood that a physical bridge system has a spring coefficient associated with these supports. However, in numerical models, these supports are often assumed to be infinitely stiff as the vertical movement in the supports is very small, relative to the vertical deflections associated with the bridge deck bending. However, bridge rotation instrumentation (DC accelerometers) is commonly deployed near the supports as maximum rotation values are observed at the ends of spans, i.e., over the supports. Consequently, these sensitive instruments detect these small vertical movements at the supports. Hence, in this work, we are specifically interested in modelling the dynamics experienced by an accelerometer placed near the supports. Therefore, to model this behaviour more accurately, we have used elastic spring supports to represent this boundary condition better. The stiffness coefficient, $k_{b}$, was assigned to these elastic supports from a commercially available elastomeric bearing’s datasheet, which met this structure’s design requirements. A damping ratio of 5% has been used.

**Figure 3:**
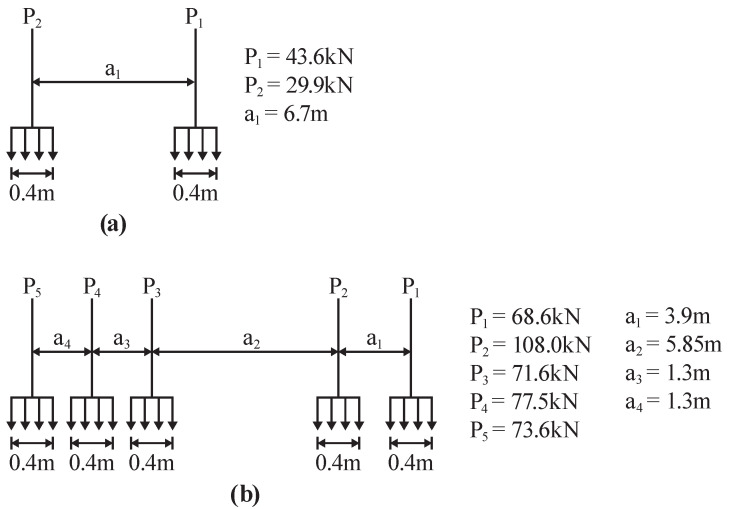
Sketch showing details of the vehicle loads used in the simulations for (**a**) two-axle vehicle and (**b**) five-axle vehicle.

To demonstrate typical end of span rotations, two vehicles are simulated crossing the bridge at $10\;m/s^{-1}$, namely: (1) a two-axle vehicle with a gross vehicle weight (GVW) of 15 t and (2) a five-axle vehicle with a GVW of 44 t. The two vehicles enter the bridge at 2 s and 8 s, respectively. To allow for the fact that the vehicle axle loads are applied via tyres, each axle load is applied as a patch load, 0.4 m long, in line with BS EN 1991-2:2003 [23]. The axle weights and spacings typical of a two-axle and five-axle vehicle are shown in Figure 3a,b, respectively. The time step used for the simulations was 1 ms.

**Figure 4:**
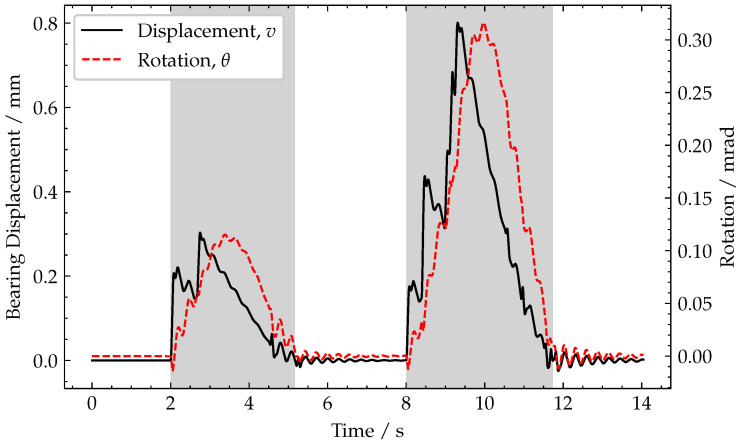
Plot of end of span rotation and end of span vertical (bearing) displacement. Periods when a vehicle is crossing the bridge are shaded grey.

Figure 4 shows that the peak vertical displacements, *v*, at the end of the deck due to compression of the bearing are 0.30mm and 0.79 mm, respectively, for the two trucks. For comparison, the corresponding peak midspan displacements due to these vehicles are 1.17 and 3.18 mm, respectively. The rotation, θ, at the end of the beam due to beam deflection is also plotted in Figure 4.

The peak rotations for each truck are 0.12 mrad and 0.34 mrad, respectively, and occur slightly after the peak bearing displacement. This offset is present because the maximum bearing displacement occurs when the load is over the bearing, whereas the maximum rotation occurs with the load is near the mid-span. After each truck leaves the bridge, a short period of damped free vibration is observed.

### 3.2. Accelerometer Model

**Figure 5:**
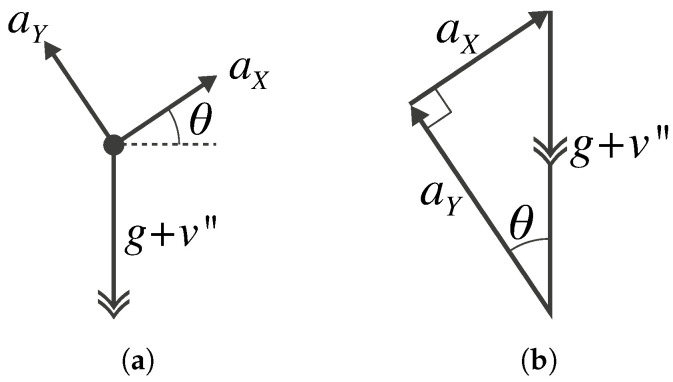
Sketches of (**a**) free-body diagram showing the apparent rotation of the Earth’s gravity (*g*) and vertical bridge acceleration ($v^{\prime \prime }$) components; (**b**) equivalent vector diagram showing the x- and y-axis acceleration components.

For ease of explanation, the vertical and horizontal axes of the accelerometer are assumed to be aligned with the corresponding global axes prior to the load arriving on the bridge. The measured accelerations comprise two components, which are illustrated in Figure 5, namely: (1) the vertical displacement of the deck, *v*, as it undergoes loading and (2) the apparent rotation by the angle, θ, of the gravity vector, *g*, and bridge deck acceleration, $v^{\prime \prime }$, in the accelerometer’s reference frame.

The following relationship is then obtained for the noise-free acceleration observed by the accelerometer:

(8) $$\begin{array}{c} a=\left[\begin{array}{c} a_{X}\\ a_{Y} \end{array}\right]=\left[\begin{array}{c} \left(g+v^{\prime \prime }\right)\sin\theta \\ \left(g+v^{\prime \prime }\right)\cos\theta \end{array}\right]. \end{array}$$

**Figure 6:**
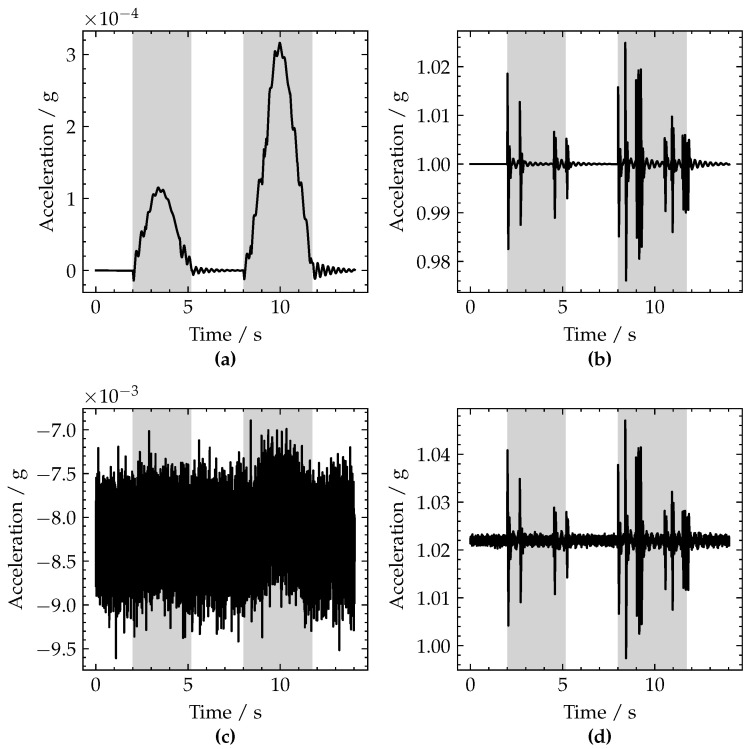
Plots of ideal accelerometer signals for (**a**) x-axis and (**b**) y-axis; simulated accelerometer signals with additive noise for (**c**) x-axis and (**d**) y-axis. Periods when a vehicle is crossing the bridge are shaded grey.

Figure 6a,b demonstrate these ideal *x* and *y* acceleration signals, respectively, using the end of span rotation and vertical displacement data shown in Figure 4.

To allow for a realistic level of signal noise, acceleration data were measured using a JAE-70SA tri-axial accelerometer at rest on a solid concrete slab floor to minimise external vibrations. From these data, the means, $\mu ={\left[\mu_{X},\mu_{Y}\right]}^{⊤}$ and covariance matrix, Σ, were calculated. The noise is simulated as a multivariate Gaussian distribution, n∼N(μ,Σ). A cross-axis sensitivity, $S_{XY}=S_{YX}=2\%$, has been assumed. Therefore, the simulated accelerometer signals, r, are given by:

(9) $$r=S_{}a+n.$$

These simulated x-axis and y-axis accelerometer signals can be seen in Figure 6c,d respectively, wherein the effect of the additive noise and cross-axis sensitivity can be observed, particularly in the x-axis signal (Figure 6c).

## 4. Numerical Simulations and Results

This section shows the effect of varying sliding window lengths on vehicle detection, as well as demonstrating the effect of sensor location on our generalised variance event detection, in Section 4.1 and Section 4.2, respectively. Section 4.3 demonstrates the approach’s robustness to the potential existence of damage in the bridge.

### 4.1. Sliding Window Length

**Figure 7:**
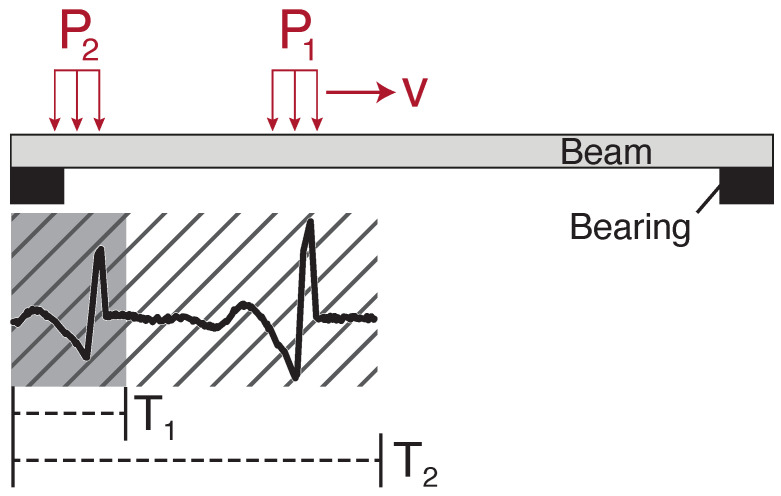
Sketch of impact loading caused by entry of vehicle axles.

The generalised variance is computed over a sliding rectangular window of length, $T_{w}$, to detect the increase in generalised variance during vehicle crossing events. This method exploits the impact loading as each axle enters the bridge (see the relatively large spikes observed when vehicles’ axles enter the bridge in Figure 6d). As the vehicle leaves the bridge, a similar effect occurs, but this time arising from the sudden unloading of the beam and bearing as each axle leaves the bridge. In Figure 7, the impulses for two axles (modelled as patch loads), $P_{1}$ and $P_{2}$, moving at constant velocity, *v*, are shown. Therefore, a sliding window of temporal length, $T_{w}$, contains the signal related to a spatial length, $L_{w}=v\;\cdot \;T_{w}$, travelled by the moving loads. This allows the choice of sliding window length, $T_{w}$, to be made based on the vehicle speed limit of the road crossing the bridge and the typical vehicle geometries encountered. As axle spacings are typically no less than 1.3 m, setting $L_{w}=0.5m$ would allow the full patch load to be captured but not multiple axles, as illustrated by $T_{1}$ in Figure 7.

**Figure 8:**
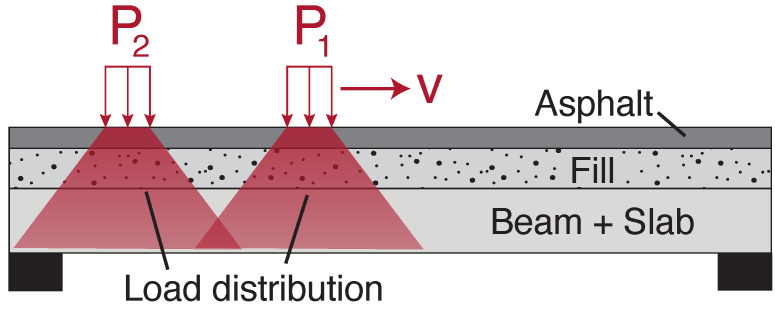
Sketch of vehicle load distribution through a bridge deck.

However, in reality this approach of setting $T_{w}$ to capture individual axles may be problematic for bridges with a larger deck thicknesses, as the load will distribute through the structure. This may be due to a number of factors including: beam depth, slab thickness, presence of fill or asphalt thickness. This is illustrated in Figure 8, where it can be seen that the applied load at the asphalt surface is distributed both vertically and horizontally through the layers of the deck. Unless the axles are sufficiently far enough apart, given the effect of load distribution, a shorter window will likely capture groups of axles and not individual axles. Therefore, a longer window (shown as $T_{2}$ in Figure 7) intended to capture a full vehicle may be employed. As the shortest European lorry wheelbases are approximately 4 m, and the longest are around 13.5 m, a reasonable sliding window length to capture full vehicles is found by setting $L_{w}$ in the range of 8m to 10m long. If a longer window was employed the detector may struggle to distinguish two vehicles with a short headway versus one longer vehicle.

**Figure 9:**
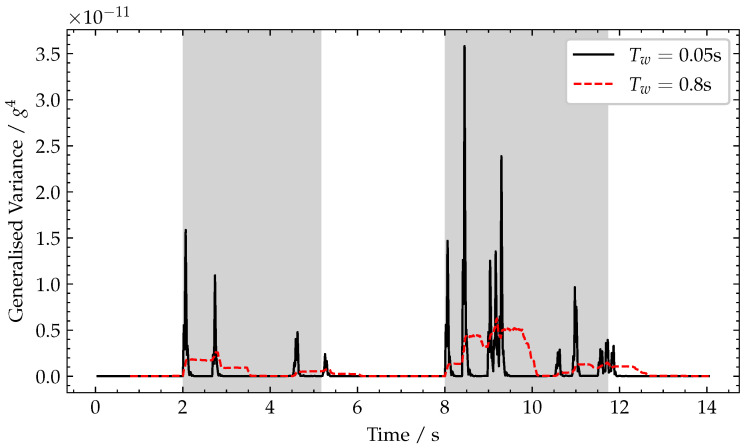
Plot of generalised variance for varying window lengths, $T_{w}$. Periods when a vehicle is crossing the bridge are shaded grey.

At 10 m/$s^{-1}$, this gives a window length $T_{w}=0.05\;s$ for the shorter window ($L_{w}=0.5\;m)$ to capture axles and a window length $T_{w}=0.8\;s$ for the long window ($L_{w}=8\;m$) to capture vehicles. To illustrate these, the data from Figure 6c,d are processed using these window lengths and are shown in Figure 9. For the shorter window length, $T_{w}=0.05\;s$, it can be noted that sharp peaks in generalised variance are seen as each axle enters and leaves the bridge. Furthermore, the relative height of these peaks corresponds to relative axle weights. For the longer window length, $T_{w}=0.8\;s$, a wider peak of lower magnitude is observed for each vehicle entry and exit. As the main focus of this paper is detecting vehicles, longer sliding windows to capture whole vehicles, i.e., $L_{w}=8\;m$, are used throughout the rest of this work.

### 4.2. Effect of Accelerometer Location

**Figure 10:**
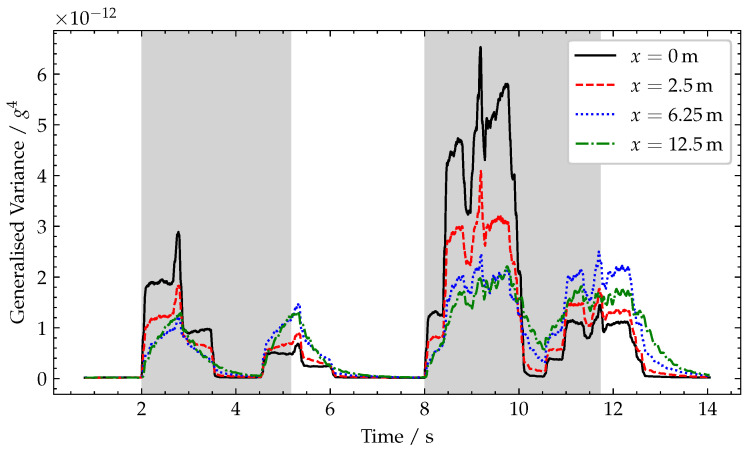
Plot of generalised variance for varying sensor locations, *x*. Periods when a vehicle is crossing the bridge are shaded grey.

To demonstrate the effect of varying sensor location, the simulated accelerometer is placed at (a) directly over the support, x=0 m, (b) close to the end of span, x=2.5 m, (c) quarter-span, x=6.25 m, and (d) mid-span, x=12.5 m. The results of these simulations are shown in Figure 10, from which it can been noted that, at all sensor locations, the generalised variance has approximately the same profile, but this is larger closer to the end of the spans, where the peak rotation values are observed.

### 4.3. Robustness to Potential Bridge Damage

**Figure 11:**
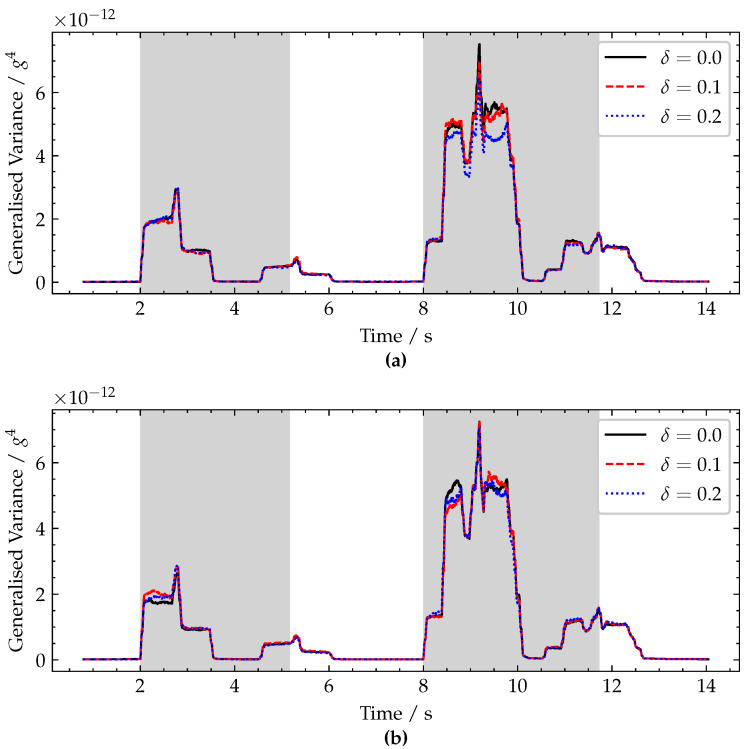
Plot of generalised variance for varying damage severity, δ, with the damage located at (**a**) mid-span and (**b**) quarter-span. Periods when a vehicle is crossing the bridge are shaded grey.

To demonstrate that the proposed approach is not sensitive to the potential presence of localised damage in the bridge, a simulation was carried out for both the healthy bridge and two separate damaged bridge cases. Specifically, localised damages are simulated at the midspan and quarter span for two different damage severities. Damage is modelled using the approach proposed by Sinha et al. [24], who represent damage severity as a ratio of crack height to beam depth, denoted by δ. Damage severities, δ=0.1 (27.1% localised stiffness loss) and δ=0.2 (48.8% localised stiffness loss), are modelled in line with what is often quoted in the literature. These damages are introduced at both the mid-span (Figure 11a) and quarter-span (Figure 11b). The results of these simulations (Figure 11) show that even these relatively large damages have negligible impact on the detection capability of the approach as the sharp rises in generalised variance when a vehicle enters the bridge are the same regardless of the damage state.

## 5. Field Trials

To demonstrate the application of our proposed event detection method, two case studies have been carried out on in-service bridges. Each case study is presented individually in Section 5.1 and Section 5.2, wherein a brief description of the structure, instrumentation and testing is provided, along with the results of applying our event detection method to the field trial data.

### 5.1. Case Study 1: Short Span Beam and Slab Bridge

**Figure 12:**
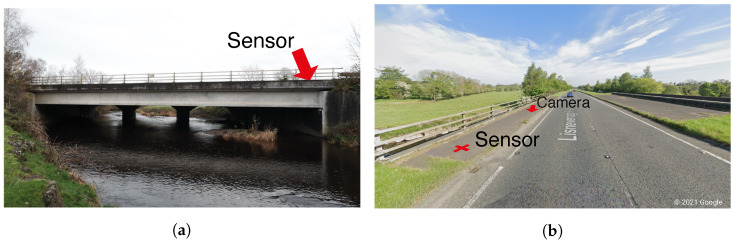
Photographs of (**a**) West elevation of bridge and (**b**) Northbound approach to bridge showing sensor and camera locations.

This field trial was carried out on the bridge shown in Figure 12. The bridge is a single 34 m span, pre-cast, reinforced concrete beam and slab bridge, which carries the Northbound carriageway, whilst the Southbound traffic is carried separately on an adjacent masonry arch bridge. For the test, a single JAE JA70-SA triaxial MEMS accelerometer was used to measure the rotation at the Southern end of span (where the Northbound traffic enters the bridge), as seen in Figure 12a. The sampling frequency (1652 Hz) was chosen based on the minimum available on the NI 9234 DAQ used for the test.

The accelerometer was placed on the pedestrian pavement adjacent to carriageway, as indicated in Figure 12b, so as to be located approximately over the web of the bridge’s edge beam. The camera was positioned at the Northern end of the bridge to record the vehicles crossing the bridge. However, unfortunately, a heavy rain shower during the testing resulted in water droplets partially obscuring the lens.

**Figure 13:**
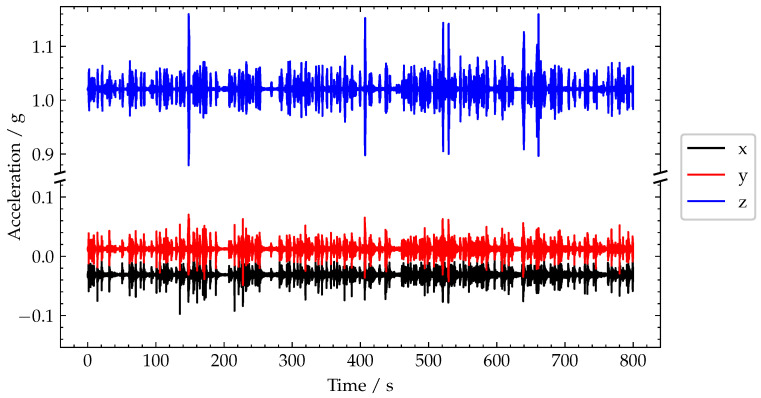
Plot of recorded acceleration signals.

In Figure 13, the raw acceleration signals recorded during an 800 s test window are plotted on a broken axis to maintain the observed biases. As the test was carried out during a relative busy period, although still with free-flow traffic, the raw acceleration time series contains a large number of transient signals, corresponding to vehicles crossing the bridge. However, the majority of these transient signals are approximately the same magnitude and thus are not very helpful to directly separate vehicle loading by some simple thresholding on the raw acceleration signals.

**Figure 14:**
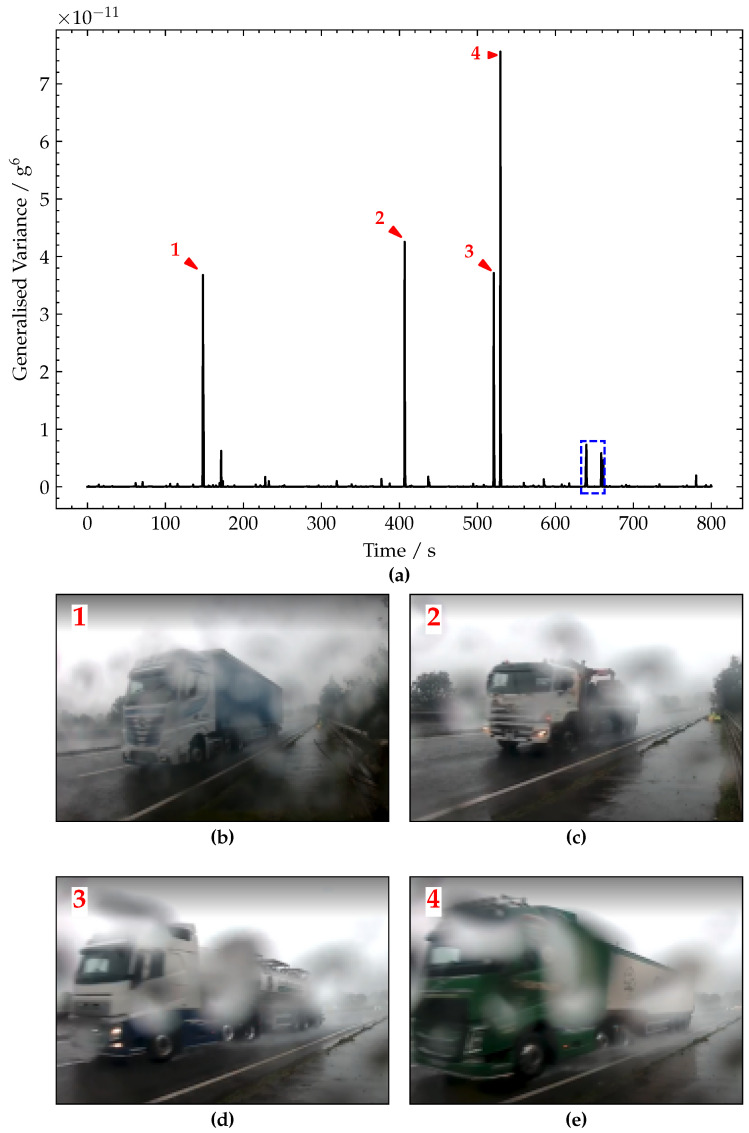
(**a**) Plot of generalised variance signal. (**b**–**e**) Pictures of vehicles corresponding to peaks 1–4, respectively.

The generalised variance of the raw acceleration signals is plotted in Figure 14a using a window length, $T_{w}=0.4\;s$ (based on $L_{w}=8\;m$ and with a free flow traffic speed of $v=20\;m/s^{-1}$). From the generalised variance plot, it is much easier to distinguish vehicle crossings, and in particular, the crossings related to the heavier vehicles, i.e., trucks. As heavier vehicles give rise to larger-magnitude structural responses, these have a better signal-to-noise ratio. Therefore, heavier vehicle events provide more information (compared with lighter vehicles events) to the subsequent SHM damage detection algorithms and help to more reliably detect bridge damage. Despite a number of large transients of similar magnitudes in the raw acceleration signals, only four of these give rise to the large generalised variance peaks which are annotated as 1 to 4. These peaks 1–4 were caused by heavy lorry crossings, as expected given their large magnitude, and frame grabs of each of these are shown in Figure 14b–e, where these are a five-axle container truck, a four-axle grab lorry, a five-axle milk tanker and a six-axle bulk feed lorry, respectively. The magnitudes of these peaks are approximately 3.7 × 10^−11^ g^6^, 4.2 × 10^−11^ g^6^, 3.6 × 10^−11^ g^6^ and 7.5 × 10^−11^ g^6^, respectively.

**Figure 15:**
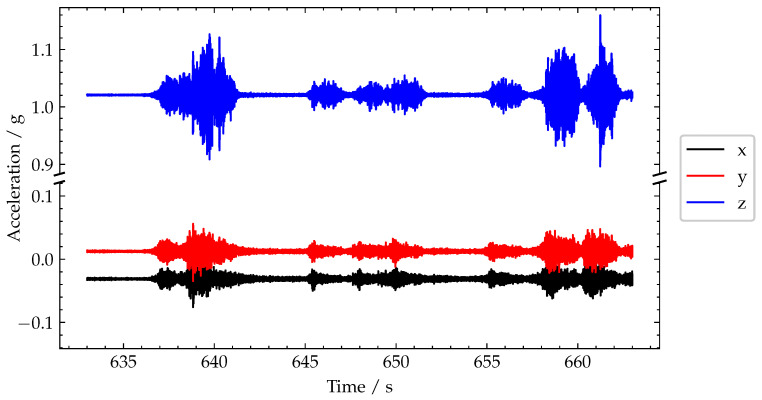
Plot of recorded acceleration signals.

To further investigate the generalised variance, and in particular some of the smaller magnitude peaks present in the time series, a zoomed-in view of the region in the blue dashed box at 635 s to 665 s in Figure 13 has been presented in Figure 15. From initial observation of the raw acceleration signals in Figure 15, approximately seven crossings are represented in this 30 s window.

**Figure 16:**
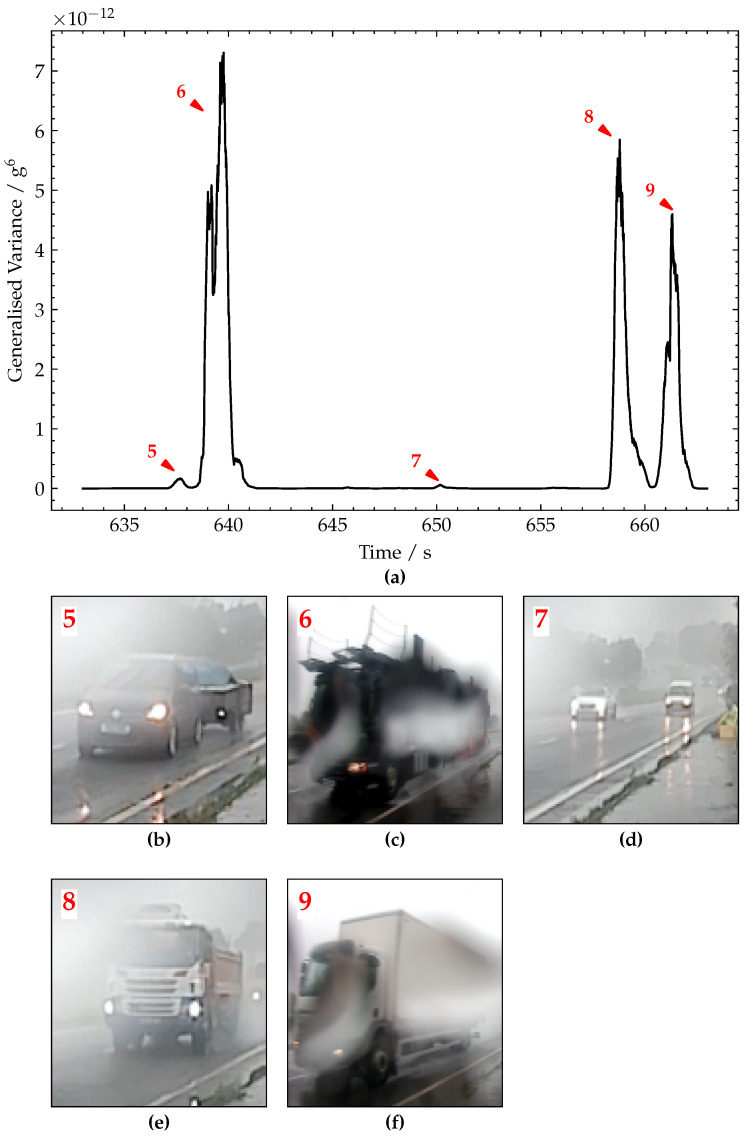
(**a**) Plot of generalised variance signal. (**b**–**f**) Pictures of vehicles corresponding to peaks annotated 5–9, respectively.

However, in the generalised variance plot (Figure 16a), only five of these lead to discernible peaks—indicating that two of the seven pulses were due to very light vehicles. Of the five discernible peaks, only three are significant (annotated are 6, 8 and 9). Frame grabs of the lorries causing these responses are shown in Figure 16c,e,f showing an almost empty five-axle car transporter, a four-axle tipper lorry and a two-axle truck, respectively. The magnitudes of these peaks are approximately 7.1 × 10^−12^ g^6^, 5.7 × 10^−12^ g^6^ and 4.5 × 10^−12^ g^6^, respectively. These smaller magnitudes (relative to the height of the peaks in Figure 14b) are in line with the smaller/lighter trucks which caused these responses.

The smaller peaks in Figure 16b correspond to even lighter-weight vehicles. The peak labelled 5 was caused by a car and trailer (Figure 16c). Furthermore, the peak annotated as 7 was actually caused by several cars and a van crossing at the same time (shown in Figure 16e). Comparing Figure 16 to Figure 15 shows the key advantage of using the generalised variance versus using the peaks-over-threshold approach applied to the raw acceleration signal. Specifically, if one only had Figure 15 to work with, firstly, it would not be obvious what threshold to set. However, more importantly, it would be extremely difficult to accurately distinguish between truck loading events and car loading events. This distinction is especially important for bridge SHM, as cars are typically not heavy enough to generate significant deformations in the bridge. Hence, the deformation response signals due to cars often contain little information useful for bridge SHM, in particular, approaches that are interested in the static component of the bridge response, e.g., maximum displacement or maximum rotation. Therefore, avoiding using scarce power and communications resources to record and transmit this kind low value data from car loading is advantageous.

### 5.2. Case Study 2: Medium-Span Tied Arch Bridge

**Figure 17:**
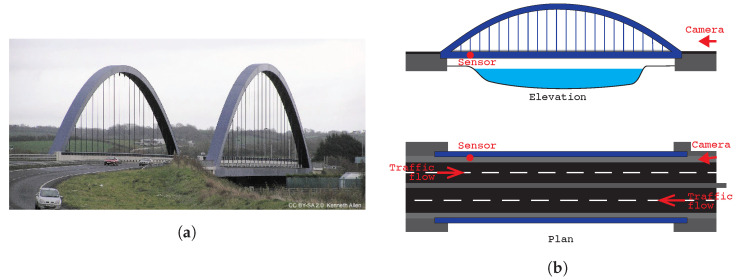
(**a**) Photograph of bridge from the western aspect and (**b**) test setup site elevation and plan.

In this case study, a different bridge and sensor were used to check that the proposed approach is not overly sensitive to the bridge being monitored or the instrumentation used. This field trial was carried out on the bridge shown in Figure 17a. This is a single-span tied arch bridge, with a span length of 98.8 m.

The instrumentation used in this test was based on a Kionix KXRB5-2050 tri-axial MEMS accelerometer, recorded at a sampling frequency of 256 Hz. This accelerometer has a significantly lower unit cost at approximately USD 4 each versus over USD 1000 each for the JAE accelerometers used in the previous case study. This is reflected in the performance of the accelerometer itself with a data sheet specified noise density of 45 μg/$\sqrt{\mathrm{Hz}}$ compared to 1 μg/$\sqrt{\mathrm{Hz}}$ for the JA70-SA.

For this test, the accelerometer was approximately 8.5 m in from the northwestern end of the span, placed on the pavement beside the parapet, as indicated in Figure 17b. A camera was placed at the eastern end of the bridge to record the traffic on both carriageways crossing the bridge during the test.

**Figure 18:**
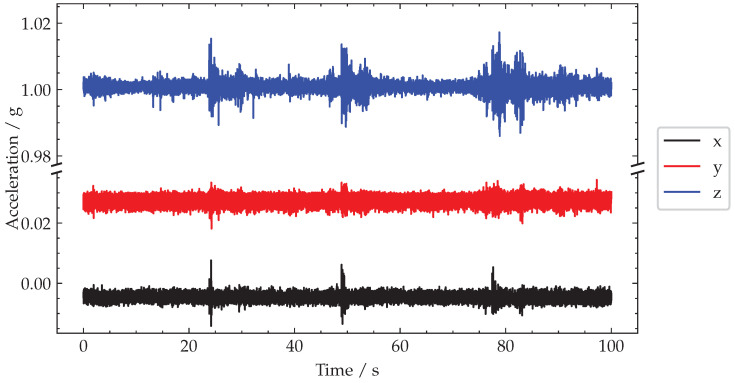
Plot of recorded acceleration signals.

In Figure 18, the raw acceleration signals recorded during a 100 s test window are plotted on a broken axis to maintain the observed biases. A sliding window of $T_{w}=0.45\;s$ was used based on a spatial length to observe $L_{w}=8\;m$ and the traffic velocity, $v=17.8\;m/s^{-1}$.

**Figure 19:**
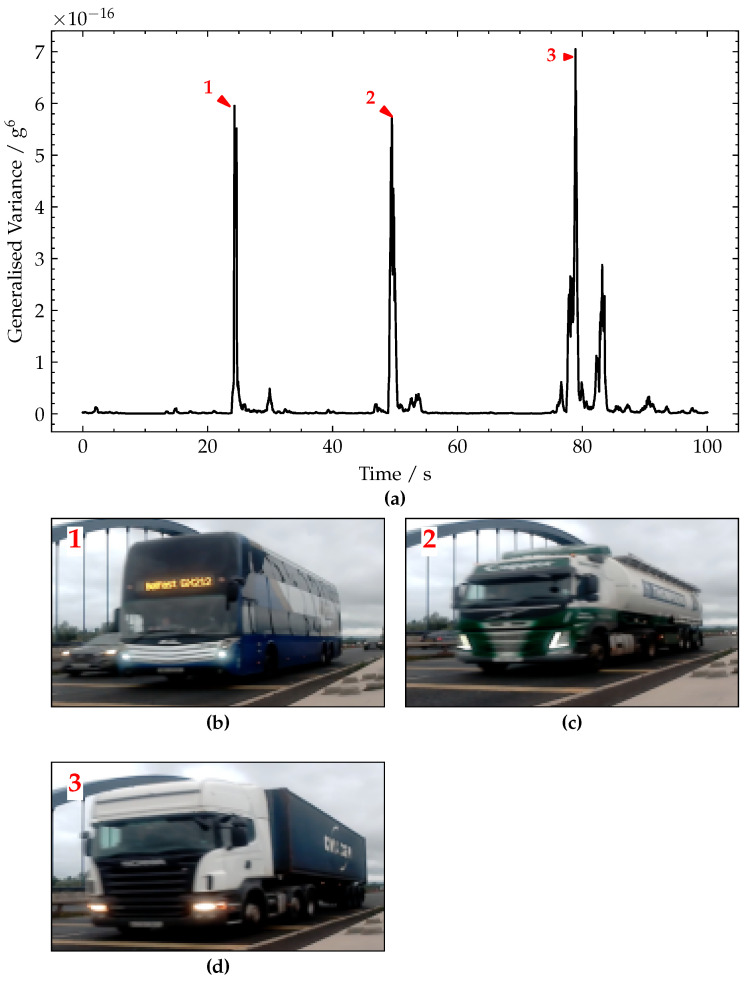
(**a**) Plot of generalised variance signal. (**b**–**d**) Pictures of vehicles corresponding to peaks annotated 1–3, respectively.

The generalised variance signal is shown in Figure 19a. From this plot, three distinct peaks are apparent, labelled 1–3 for convenience with the frame grabs from the corresponding video footage shown in Figure 19b–d. These peaks 1–3 were caused by heavy vehicle crossings, specifically: a three-axle double-decker bus, a six-axle bulk feed lorry and a five-axle container lorry. The approximate magnitudes of these major peaks are 5.9 × 10^−16^ g^6^, 5.7 × 10^−16^ g^6^ and 6.9 × 10^−16^ g^6^, respectively. Each crossing appears as a pair of peaks, corresponding to vehicle entering and exiting the bridge, respectively.

The results shown in Figure 19 show that the proposed approach was not sensitive to the bridge monitored or the sensor used, as it performed equally well on a second bridge using a significantly lower-grade sensor.

## 6. Discussion

The results of the numerical simulations (Section 4) show that the proposed approach is relatively insensitive to sensor placement and is robust to the potential presence of damage in the bridge. Field trials (Section 5.1 and Section 5.2) have demonstrated the successful application of the proposed detector on two different classes of bridge structure with different qualities of accelerometer. Not only was the proposed detector able to indicate the temporal regions of interest related to vehicle crossings, but the magnitude of the generalised variance allowed events due to heavy vehicles, such as trucks, to be distinguished.

Being able to accurately determine events caused by heavy vehicles is valuable to bridge SHM approaches focused on bridge static response, as for the most part, only heavy vehicle loading produces interpretable bridge-response signals. Therefore, the proposed detector can be used to identify at the source the more valuable periods of data by reliably distinguishing truck events from car events or noise. This allows the SHM system to only record and process the data during these more valuable events and can enable more efficient BSHM deployment in environments with scarce power and communications resources.

A detection system that could identify axles is preferable for bridge SHM, as opposed to one that identifies vehicles, as knowing the number of axles would go a long way towards classifying the vehicle. However, as discussed in Section 4.1, making the windows short enough to try and detect individual axles may be thwarted by the distribution of axle loads through the asphalt and the deck, particularly for closely spaced axles. Hence, this study focused on detecting vehicles. However, in future work, data will be collected from a range of different bridges to determine if axle detection can be reliably achieved on typical bridges.

The current work was envisaged for short to medium-span bridges where, most of the time, only one truck at a time will be present on the bridge. The issue of detecting multiple trucks on a bridge at the same time is something that could be explored in further work. Moreover, there seems to be two likely angles of attack to this challenge. The first is to move the burden of detecting multiple vehicle events to a computer vision system, and this will be most helpful for trucks travelling side by side, i.e., one truck in the fast lane and one truck in the slow lane. However, if one was more interested in detecting consecutive trucks in the slow lane, then a combination of both optimising the sliding window lengths and possibly adding another detector at the downstream end of the bridge could achieve this. Specifically, with the additional sensor, both the entry and exit times for the vehicles could be reconciled to determine the crossings, particularly for longer individual spans, e.g., span lengths greater than 50 m.

## 7. Conclusions

This paper presented a vehicle detection method based on the generalised variance computed over a sliding window on raw bridge acceleration data. This obviates the need for additional bridge instrumentation and is achieved with minimal computation requirements. It was shown that the sliding window length can be chosen based on the typical traffic speeds on the bridge being instrumented and that the detector is relatively insensitive to sensor placement on the bridge. Field trials on two bridges showed the effectiveness of the proposed detector at identifying vehicle crossing events, and that these could be discriminated not only temporally into regions of interest but also by the magnitude of the generalised variance. Distinguishing events based on the magnitude of the generalised variance allows the accurate detection of loading events caused by heavy vehicles such as trucks.

## Data Availability

Not applicable.

## References

[1] Brownjohn J.M.W., Aktan E. Improving Resilience of Infrastructure: The Case of Bridges. Proceedings of the Structures Congress 2013: Bridging Your Passion with Your Profession—Proceedings of the 2013 Structures Congress.

[2] Cheilakou E., Tsopelas N., Anastasopoulos A., Kourousis D., Rychkov D., Gerhard R., Frankenstein B., Amditis A., Damigos Y., Bouklas C. (2018). Strain Monitoring System for Steel and Concrete Structures. Procedia Struct. Integr..

[3] Lydon D., Lydon M., Taylor S., Del Rincon J.M., Hester D., Brownjohn J. (2019). Development and Field Testing of a Vision-Based Displacement System Using a Low Cost Wireless Action Camera. Mech. Syst. Signal Process..

[4] Khan M.A., McCrum D., Obrien E.J., Bowe C., Hester D., McGetrick P.J., O’Higgins C., Casero M., Pakrashi V. (2022). Re-Deployable Sensors for Modal Estimates of Bridges and Detection of Damage-Induced Changes in Boundary Conditions. Struct. Infrastruct. Eng..

[5] González A., Hester D. (2013). An Investigation into the Acceleration Response of a Damaged Beam-Type Structure to a Moving Force. J. Sound Vib..

[6] Huseynov F., Kim C., OBrien E.J., Brownjohn J.M.W., Hester D., Chang K.C. (2020). Bridge Damage Detection Using Rotation Measurements—Experimental Validation. Mech. Syst. Signal Process..

[7] Riches O., Hill C., Baralos P. (2019). Queensferry Crossing, UK: Durability, Maintenance, Inspection and Monitoring. Proc. Inst. Civ. Eng.—Bridge Eng..

[8] Yan Y., Mao X., Wang X., Yu X., Fang L. (2019). Design and Implementation of a Structural Health Monitoring System for a Large Sea-Crossing Project with Bridges and Tunnel. Shock Vib..

[9] Ferguson A.J., Hester D., Woods R. (2022). A Direct Method to Detect and Localise Damage Using Longitudinal Data of Ends-of-Span Rotations under Live Traffic Loading. J. Civ. Struct. Health Monit..

[10] Jeng S.T.C., Ritchie S.G. (2008). Real-Time Vehicle Classification Using Inductive Loop Signature Data. Transp. Res. Rec..

[11] Balid W., Refai H.H. (2018). Real-Time Magnetic Length-Based Vehicle Classification: Case Study for Inductive Loops and Wireless Magnetometer Sensors in Oklahoma State. Transp. Res. Rec..

[12] Gingras D.J., Corriveau R.J.L., Soileau M.J., Auger M. (1998). Optics and Photonics Used in Road Transportation. Lasers and Materials in Industry and Opto-Contact Workshop.

[13] Seo Y.G., Lew C.G., Lee B.H. (2016). Development of Vehicle Classification Algorithm using Non-Contact Treadle Sensor for Toll Collect System. J. Korea Inst. Electron. Commun. Sci..

[14] Won M., Zhang S., Son S. WiTraffic: Low-cost and Non-Intrusive Traffic Monitoring System Using WiFi. Proceedings of the 2017 26th International Conference on Computer Communications and Networks, ICCCN 2017.

[15] Ma R., Zhang Z., Dong Y., Pan Y. (2020). Deep Learning Based Vehicle Detection and Classification Methodology Using Strain Sensors under Bridge Deck. Sensors.

[16] Kleyko D., Hostettler R., Lyamin N., Birk W., Wiklund U., Osipov E. Vehicle Classification Using Road Side Sensors and Feature-Free Data Smashing Approach. Proceedings of the 2016 IEEE 19th International Conference on Intelligent Transportation Systems (ITSC).

[17] Gerek O.N., Ece D.G., Barkana A. (2006). Covariance analysis of voltage waveform signature for power-quality event classification. IEEE Trans. Power Deliv..

[18] Wang T., Qiao M., Zhu A., Niu Y., Li C., Snoussi H. (2018). Abnormal Event Detection via Covariance Matrix for Optical Flow Based Feature. Multimed. Tools Appl..

[19] Key S.C., Smithson S.B. (1990). New Approach to Seismic-reflection Event Detection and Velocity Determination. Geophysics.

[20] Wilks S.S. (1932). Certain Generalizations in the Analysis of Variance*. Biometrika.

[21] Horn R.A., Johnson C.R. (2012). Matrix Analysis.

[22] Marcus M., Minc H. (1992). A Survey of Matrix Theory and Matrix Inequalities.

[23] British Standards Institution (2010). BS EN 1991-2:2003 Eurocode 1: Actions on Structures—Part 2: Traffic Loads on Bridges.

[24] Sinha J.K., Friswell M.I., Edwards S. (2002). Simplified Models for the Location of Cracks in Beam Structures Using Measured Vibration Data. J. Sound Vib..

